# Sounding the Archival Silence: Searching for Music in the Nineteenth-Century English Asylum

**DOI:** 10.1093/shm/hkac035

**Published:** 2022-08-05

**Authors:** Rosemary Golding

**Keywords:** Archives, asylum history, music history, music therapy, mental health

## Abstract

The music of the nineteenth-century English asylum provides a rare insight into the place of music within the structure of a medical institution during this period. Yet with archives literally ‘silent’, how far can the sound and experience of music be retrieved and reconstructed? Drawing on critical archive theory and the idea of the soundscape as well as musicological and historical practice, this article questions how we can investigate asylum soundscapes through the silences of the archive, and how we can use the resulting processes to deepen our relationship with the archive and enrichen other aspects of historical and archive studies. I argue that in drawing attention to new forms of evidence in order to address the literal ‘silence’ of the nineteenth-century asylum, new approaches to metaphorical ‘silences’ can be found.

Archives and sound do not tend to mix. Archivists, and users of archives, will be familiar with the hushed presence of the document room, the occasional hum of a photocopier, swish of a door or quickly-silenced bleep of a mobile phone. Yet musicologists active in archives are in search of exactly those noises and musics of the past which are so hastily muzzled in the modern context. Archival materials are an essential tool in the musicological toolbox of the twenty-first century, offering an invaluable resource for any scholar seeking to understand how music was discussed, written about, performed and listened to in past eras.^1^ The music of the nineteenth-century English asylum is no exception, with archives providing a rare insight into the place of music away from the concert hall and among ordinary people, within largely closed communities. It also offers an insight into the soundworlds of patients at asylum institutions, and particularly the use and location of music as part of a therapeutic package. Investigating the music of the asylum also helps illuminate more general historical details, such as elements of gender and class, patient activity and staff employment. Yet with archives literally ‘silent’, how far can the sound and experience of music in historic asylums be retrieved and reconstructed? And how do we go beyond this to look to fill the other ‘silences’ of the asylum archive: the gaps in documentation, the voices absent from representation, the experiences lost to the past? Addressing these silences draws on the full range of the archive’s offering and beyond, read critically and creatively. Drawing on critical archive theory as well as musicological and historical practice, I ask how we can investigate asylum soundscapes through the silences of the archive, and how we can use the resulting processes to enrich other aspects of historical and archive studies.

Asylum history has long been a successful focus for historians and archivists alike.^2^ With the exception of detailed investigations by Australian historian Dolly McKinnon, the sonic and musical world of the asylum has received little attention; notably, recent texts such as Jennifer Wallis’s *Investigating the Body* have largely omitted aural and musical experience from their purview.^3^ Yet sound and music certainly contributed to contemporary experiences of the asylum. John Conolly describes eighteenth-century asylums, where local people ‘walked far round, to avoid hearing the cries and yells which made night hideous’; Conolly’s recommendations for the practice of moral management and non-restraint include space for quiet, as well as provision of music.^4^ Many public institutions, closed in the second half of the twentieth century, are memorialised in vast archival collections spanning official documents, printed memorabilia and ephemera dating back to their foundations. State pauper asylums largely date from the period between 1808 and 1850; the 1808 Asylum Act allowed local authorities to use public funds to build and maintain a pauper asylum, while the 1845 Act made this provision obligatory. By the turn of the twentieth century nearly 100,000 patients were resident across over 100 asylums in the United Kingdom, each carefully regulated and monitored. The exponential growth of the pauper lunatic population meant that many asylums housed over a thousand patients, and counties such as Surrey and Yorkshire operated multiple institutions spread over several sites.

The Victorian drive for centralisation, standardisation and regulation meant that the asylum project was accompanied by a mass of paperwork. Parliamentary bills and reports, formal records from the institutions themselves, patient case notes, the reports of the overseeing Commissioners in Lunacy, and the minutiae of meeting minutes, correspondence, accounts and patient communications, form part of the thick seam of material successfully mined by historians of medicine and society. In addition, asylum officers (in particular, the Medical Superintendents with responsibility for the day-to-day running of each institution) produced their own paper trail. Frequent correspondence, visiting, and publishing, from the 1840s under the auspices of the Association of Medical Officers of Asylums and Hospitals for the Insane, offered numerous outlets for medical specialists to share their views on asylum management, and the causes and treatment of insanity.

Music became embedded in English lunatic asylums via the practice of moral management, a theory that engaged all elements of patient life—from diet and clothing to exercise and work—in careful balance designed to foster calm and self-control.^5^ Many state pauper asylums introduced employment for patients largely as a means to keep them occupied, as well as to help with the always-precarious financial situation of institutions funded by local taxes. At most asylums, opportunities for recreation followed soon afterwards, both to cater for patients unable to engage in manual work and to help occupy the few hours in the evenings and on Sundays when work was prohibited. From the introduction of books and outside exercise, entertainment grew to include sports, dancing, theatre and concerts.^6^ The provision of recreation halls and facilities for music and dance was often recommended by the Commissioners in Lunacy.^7^ Music was also recommended in contemporary writings on lunacy and moral management. George Man Burrows (1771–1846), for example, wrote in 1828 of music’s reputation as ‘not only … an amusement for the insane, but as a powerful means of cure’, while John Conolly (1794–1866) recommended in 1847 that ‘the attendants should be encouraged to learn singing and music, so that their means of amusing the patients may be increased’.^8^

By the 1870s it was usual to find a weekly dance, accompanied by the asylum band^9^ composed of musically-trained attendants, together with visiting performers, a well-stocked library, bird cages, landscaped grounds, sports teams and regular fairs and celebrations. Although music was widely used and recognised for its therapeutic qualities in asylums, it was never formally accepted by the wider scientific community; attempts to undertake experiments in music’s efficacy as treatment by the Guild of St Cecilia in the 1890s were received with scepticism.^10^ Music and other forms of recreation helped demarcate the asylum’s daily and weekly schedule, structure social gatherings (particularly those between men and women) and offer a reward for good behaviour. They also formed a link between the closed world of the asylum and the patterns of day-to-day life and behaviour in the world outside.^11^ Within the private and charitable asylums, smaller patient numbers and the presence of musically-trained and -equipped patients (and staff) led to the inclusion of musical activities better-suited to middle- and upper-class patients, such as soirées and chamber music concerts.^12^ The impression given in the regular Reports produced by each institution, as well as in newspaper reports and contemporary literature, is often one of a social whirlwind, in which patients moved from one enjoyable activity to another.^13^ Yet how accurate is this picture in filling out the archival silence which has surrounded music and the soundworld of the asylum?

Before continuing I would like to add a brief note regarding terminology. Many of the terms, ideas and labels used in the nineteenth century are uncomfortable or inappropriate for modern times. Nineteenth-century asylums and other institutions offering care and residence for patients experiencing poor mental health worked using both outdated ideas, and experimental treatment, but often had little consideration for the patient as an individual, particularly within the growing public institutions. In this article ‘mental health’ is used as a term to denote the broad span of conditions and symptoms presented by patients, ranging from chronic and severe psychological illnesses to short-term stress, anxiety, malnutrition or depression. The kinds of documents, materials and archives we use inevitably affect the ways in which we consider patient treatment, and the impressions and judgments we (subconsciously or not) form about asylum history. A focus on music, entertainment and therapeutic activity serves to draw attention away from some of the unimaginable horrors of asylum life and patient experience. Thus it is important to maintain a balanced view as well as recognising changes in medical and moral contexts.

## Archival Silences and Musicological Soundings

Recent research has fostered a concern with archival ‘silences’: the gaps and spaces in the archives where individual voices and perspectives are missing, and the methods and imaginatory leaps we might take to address or recognise these. These silences can be deliberate, respectful, political and powerful.^14^ Archival practice has embraced a new creativity, seeking to bring alternative voices to the fore in order to explore well-trodden paths from new perspectives. Anne J. Gilliland and Michelle Caswell, for example, explore the affects, emotions and expectations tied up with archival materials, particularly those which are inaccessible or absent, and which might turn out to be misplaced.^15^ It does not take long working amongst the archival materials left by the Victorian asylums to begin to feel an engagement with the characters that populate narratives, but the details surrounding patients’ day-to-day existence is, in most cases, woefully documented. Silences are not just about missing details, but rather an outcome of the acts of archives and record-keeping as power production, oppression and liberation.^16^

Power and authority are particularly relevant in the world of the nineteenth-century asylum, where patients had very little autonomy. A longing for records that are lost or non-existent is associated with a desire to tell the story of the subaltern subjects and their voices missing from the historical record.^17^ The move towards a patient-centred approach to history (often termed history-from-below, developed from the 1980s by scholars such as Roy Porter) can therefore be closely connected with the desire to critique and broaden the archive and associated historical methodology.^18^ The approaches taken in recent writings within critical archival studies draw on a much longer tradition of critical history which might be traced back to the ‘historical imagination’ put forward by Robin G. Collingwood.^19^ Collingwood argued for history to maintain a distance from the ‘positivist fallacy’ that came from scientific models.^20^ He posited instead the need for a ‘constructive imagination’ to be drawn between the series of fixed points, themselves ‘achieved by critical thinking’.^21^ Scholars such as Gilliland and Caswell have drawn these concerns into a more direct focus on the ways in which archives are used and constructed, as well as the uses of history among specific groups and communities.

In a similar vein, much recent musicological work has been directed towards ‘soundings’: using new resources to seek a more detailed and critical picture of the ways in which music and sound were present and experienced in the historical and geographical distant.^22^ Musicological research has sought a more immersive way of reconstructing musical pasts, looking far beyond the musical works which have always been at the core of historical musicology, towards the deep contexts of musical performers, composers, audiences, critics, and their social and cultural spheres. Such ‘soundings’, aiming to revivify the aural world of a particular location or context, result in a rich insight into worlds often hidden when music is simply approached by works and composers. Historical work on private, domestic music-making and a wide range of institutions has benefited from this new approach with a range of creative outputs. Projects such as the ‘Listening Experience Database’ had sought to track the musical experiences of ‘ordinary people’ as a new form of understanding music’s role and effect, seeking out the kinds of evidence widely absent from historical narratives.^23^ Yet the historical presence of sound remains a challenge: as Ian Biddle and Kirsten Gibson suggest, ‘aurality, as an “object” of historical enquiry, is radically dispersed: its materials are distributed, especially in the pre-phonographic era, across numerous media, locked into the interstitial spaces between texts, images, scores, buildings and instruments (both musical and technical) for the production of sound’.^24^

Musicological ‘soundings’ are embedded in the move towards a sociological approach to music studies which has captured much of the discipline since the 1980s. This move draws heavily on ethnography as well as sociology and the growing sub-disciplines of history including economic history. The approach has been applied to studies of both text and music: Lawrence Kramer, for example, understands the term ‘sounding out’ to mean ‘read with effort by piecemeal combination, addressed in exploratory ways for alliance and mutual understanding, grasped in the forms of echoes from an unplumbed depth, and made to sound as clearly as possible against a prevailing silence’.^25^ Kramer’s key interest is in the social and cultural meanings of musical works. The meanings of the music to be explored between the pages and artefacts of the archive are equally important, with multiple perspectives belonging to patients, staff, management, overseers and local societies. In these cases, music is a more abstract phenomenon, expressed in genres and generalities rather than composers and works. In this sense musical meaning is deeply contingent and contextual. While the meanings and purposes of music in healthcare settings and institutions have no doubt changed, other continuities can be drawn from personal responses to music and its role in society and culture. Tropes regarding music’s power over the human mind and body can be traced back to ancient times as well as to modern medical work, giving particular significance to the stories of the historical archive.

The formal records from nineteenth-century asylums certainly embody an inbalance in power, particularly in the case of pauper and charitable institutions where social class added to the inequality between the sick and the well, the contained and the free. Research can only be based on what is left behind, and the ephemeral nature of much of the asylums’ day-to-day life meant that only what was considered important enough to record or preserve is left for modern scholars. For the purposes of this study, formal reports contained the most information about the use and role of music; patient case records were largely unhelpful. As Michelle Caswell, Ricardo Punzalan and T-Kay Sangwand note, ‘there is no neutral in archives’.^26^ The vast majority of the records referred to here are written by the powerful members of the asylums’ management, and the government’s Commissioners in Lunacy. Not only do we miss the individual voices of patients, we lack almost the entire perspective of the lower classes, including the staff of nurses and attendants. Records were, of course, written with a purpose, and are constructed to present a largely positive, sanitised view of the institution. The narratives of music in asylums can only ever be a partial record, particularly where the effects of music are concerned. We can, at best, explore the ways in which music as therapy was constructed, debated and theorised; many records simply tell us how it was presented, perhaps as a means for securing greater investment or a method for gathering public support.

Asylum archives include numerous statistics from the countable world of the Victorian asylum: patients admitted and cured; patients and staff attending musical events; payments made for instruments, music, or performers; concerts performed and dances organised. The world of data, numbers and facts helps to offer a framework for assessing the structure of musical activity and its worth within the organisation. In some cases the statistics are all that remain, with little or no indication of the ways in which music was perceived or experienced, and of the individuals who made up the band, the audience or the dance. The data in the case studies allow institutions to be compared, from their size to the dates attached to innovations such as the bands, choirs and recreation halls. However, it is the tangled web of institutional narratives that produces the most detailed and interesting results from which to build a sense of how music was used and valued.

A thick historical approach makes use of all the assets of the archive and beyond to investigate how characters of the past thought about and experienced music, including the performers, composers, promoters, managers, listeners and bystanders. But how possible is it to make the leap towards a fuller account of music’s presence in areas of everyday experience, to fill the archival silences that remain about music’s role, the details of its performance and the experience of its hearers, its locations and manifestations, and its position in a wider soundworld? For considering the ‘soundscape’ requires an engagement not only with the music and sound that was to be heard in the Victorian asylum, but the ways in which it formed part of a social and cultural world, its contribution to the experience of patients and staff alike, and its meanings and relevance. As John M Picker notes, the soundscape ‘requires not only sound within space but also a listener in a position to hear it’ (2019, p. 147). The leaps involved in considering the asylum soundscape in its broadest sense therefore prompt us to return to the institution and its patients in new ways.

This article considers the potential of musicological-historical methodology for addressing the archival silence in the particular case of the nineteenth-century English lunatic asylum. Asylums have long formed an important locus for historical enquiry into nineteenth-century perspectives on madness and mental illness, class, gender, institutionalisation, the role of the state, charity and philanthropy, and so on. Since the 1980s, scholars have urged the importance of finding new perspectives in order to ensure the viewpoints of patients, in particular, are heard. Since then patients’ voices, their relationships with medical staff, their letters, surroundings and everyday activities, processes of admission and release, and patients’ families and other visitors, have all come under the scholarly spotlight. Yet among the remaining archival ‘silences’, the soundworld of the asylum continues to be relatively un-investigated. Patient and staff voices can be ‘heard’ through the medium of written text, but the evidence for what was physically heard, listened to and experienced in asylums has received little attention.

## Evidence for Music in the Asylum

The Victorian asylum provides a particular context for an exploration of soundscapes and the archive. As John M Picker notes, the nineteenth century saw ‘new powers of listening’ in the development of sound and recording technology, while at the same time a host of new soundscapes developed in the factories and machines of the industrial revolution.^27^ With urbanisation, medical advances and changes in moral and philosophical attitudes, music, noise and silence took on new meanings as metaphors for wellness, social reform, morality and technological change. My research is focussed on both formal and informal examples of music as a part of the overall soundscape.^28^

The research undertaken as part of my project investigating music in lunatic asylums has been focussed on around a dozen examples, selected from both state-run pauper lunatic asylums, and private and charitable institutions. These range from some of the earliest state asylums at Norfolk and Wakefield to later examples such as the Brookwood asylum in Surrey, the Quaker Retreat near York, the upper-class exclusive Ticehurst Hospital, the small private Barnwood House in Gloucester, and the ancient foundation of Bethlem. As might be expected, archives are enormously varied. State-run institutions were required to keep a minimum level of paperwork on each patient, formal accounts, and annual reports, which give significant information on the day-to-day running of the asylum as well as its staff. Many have extremely rich archival collections beyond this, including concert programmes and posters relating to musical and theatrical performances, photos, and patient-produced ephemera. The materials left extant by private asylums are far more varied in archival practice, with formal records often limited, although some produced far more in the way of patient material and printed matter relating to music and asylum life. Of course, all such collections are dependent on the work of collectors and archivists, together with the vagaries of storage and natural disaster.

The choice of institutions for close study was influenced by geography, archive accessibility, the institution’s reputation for moral management or innovative practice, and any particular links with music. The asylum at Worcester (1852), for example, is well known for having employed the composer Edward Elgar during his formative years as bandmaster for the asylum band.^29^ Among the pauper asylums, institutions including Hanwell, West Riding and Lincoln had particular reputations for developing moral management and principles of non-restraint, identifying them as innovative in approach and practice.^30^ The West Riding Asylum near Wakefield (1818) was a particular centre for medical investigation into insanity during the 1860s and 1870s, but it was also one of the most active in music and theatre.^31^ Private and charitable asylums were, again, more varied: the oldest, Bethlem, was so renowned for its poor practices that it made its way into popular culture as ‘Bedlam’, although from its removal to St George’s Fields in 1815 (coinciding with public outrage over its practices following a Government Commission) it began to see significant reform.^32^ The York Retreat (1794) and the Holloway Sanatorium (1885) were also important centres of innovation.^33^ The former, founded under the Tuke family at the end of the eighteenth century, was promoted as a new departure in humane methods of care for the insane, while the latter offered new opportunities for the middle classes to access specialist care and accommodation. In addition to these larger institutions, numerous smaller private asylums were in operation throughout the nineteenth century, some of them descended from the earlier ‘madhouses’ of the eighteenth century and beyond.^34^

As public institutions, state-run pauper asylums were managed by a Committee of Visitors, typically local clergy and landowners who brought political and legal interest and experience, but rarely medical knowledge.^35^ In most cases their main concern was to ensure the asylum was run in accordance with regulatory requirements, at minimum cost. In many cases this meant facilities were kept to a basic level, although at most institutions a level of philanthropic benevolence, as well as a desire to promote order and moral well-being, prevailed and paupers were provided with opportunities for recreation, pleasant surroundings and sufficient space. Government overview was ensured via the Commissioners in Lunacy, a group combining medical and lay men, which travelled the country examining the asylums, their staff and patients, both giving advice and ensuring standards were maintained.^36^ Within the asylum the Medical Superintendent took overall responsibility for day-to-day running and was accountable to the Committee of Visitors. From the middle of the century the Medical Superintendent was typically a trained physician; prior to this he was often appointed with moral and religious leadership in mind, or the experience of running a smaller private institution. Other officers included deputy and visiting medical staff, a Chaplain, a bursar or clerk, and a matron; the day-to-day care of patients was undertaken by male attendants and female nurses, while larger asylums would also employ carpenters, cobblers, farm staff, cooks, maids, and other domestic and manual workers.^37^

## Formal Reports, Data and Accounts

The main sources for information on how asylums were run are the Annual Reports produced by all institutions. These usually contained reports from the Medical Superintendent, Committee of Visitors, and Chaplain, together with data on patient admissions, discharges and deaths, and summary accounts. Details of the place and role of music were frequently found within both the Superintendent’s and the Chaplain’s reports. Medical Superintendents often included a section on Recreations and Amusements, explaining the provision of books, games, sports and other activities, listing the concerts and events that had taken place, and giving data for the proportion of patients that had taken part. The Commissioners in Lunacy also commented regularly on the provision of recreations and amusements, in some cases urging Superintendents to make improvements by supplying additional resources or making new arrangements. This was particularly the case where Visitors resisted the expense of building a separate hall for recreational use.

Chaplains’ reports give further information about the inclusion of music in religious observances. Purpose-built chapels were only mandatory from the 1880s, and provision for worship varied enormously; with growing patient populations the pressure on space was acute, and religious services were found in dining halls, recreation halls and dormitories as well as dedicated buildings. The Reports give details about the installation of a harmonium or organ, the development of a choir, patient involvement in singing during the services, the choice of hymn books or choral settings, and other innovations widely pursued by Chaplains, such as singing classes.

In many examples, Superintendents and Chaplains also give an indication of the perceived, or purported, medical benefits of music-making in both secular and religious contexts. Music was widely described by Superintendents as an aid against the monotony and dullness of institutional life. This could be directly linked to therapeutic impact: for depressive patients, music was recommended as a means for improving mood and liveliness, while for manic patients attendance at a concert, dance or religious service could help use excess energy or improve self-control. Other Superintendents made more specific connections between music and an improvement in patient mental health, although individual cases are rarely explored in detail.

The idea that music could become a positive aspect in the therapeutic regime developed through these connections and the support of the Commissioners in Lunacy. Yet the evidence base for music’s efficacy is absent from the formal Reports. Formal records formed part of the regulatory machine with the oversight of the Commissioners, but also formed part of the relationship between the Medical Superintendent and the Committee of Visitors. With overall financial and moral responsibility for the institution, the Committee’s interest in the expenditure of each asylum meant that the inclusion of activities such as music, dance, or sport, needed justification as part of the medical or moral purpose. Furthermore, the formal reports formed part of the asylum’s portrayal to the local community. The use of music helped to illustrate the asylum’s position as a humane and benevolent institution, and its connection with therapy helped to justify the use of public funds and to encourage further philanthropic support. It is important to remember, then, that the descriptions of the reports are included with specific social and cultural ends in mind regarding the asylum’s management and its place in the community.

Asylum records are full of data, from patient numbers (admissions, discharges, rudimentary analyses of the causes and diagnoses of illness, physical symptoms and deaths) to financial accounts. There is plenty in these kinds of details to add to our picture of music and the soundscape of the asylum, from staff participation to musical instrument purchase. Tracking the members of an asylum band has its own challenges; at some institutions band members were paid a gratuity on occasion, which allows the number of members to be calculated; elsewhere it was just the bandmaster or instructor that was remunerated. With band pay not forming a part of the regular wages, it is not usually possible to trace band members in the formal accounts or wage records. Conversely, bandmasters and organists were rarely ranked among the regular staff of the institution and so their salaries are not included in staff lists and wage books. Bands such as the ensemble at the Worcestershire County Pauper Asylum, which was a collaboration between internal staff and visiting professionals, therefore fall across several sets of records, particularly where financial details are sought.

At other times the formal accounts offer details missing from elsewhere in the archive. The question of whether Bethlem Hospital had an organ, for example, required drawing together evidence from both formal minutes and the day-to-day ledgers. An organ is first mentioned in the earliest printed Annual Reports of 1843, when it was argued ‘it would tend greatly, it is submitted, to relieve the monotony of the service as at present performed, were an organ, which could be provided at a small cost, introduced. This would enable the chaplain to introduce psalmody … The previous habits of the patients render the omission and not the introduction of singing a novelty’.^38^ In 1845 it was reported that the chapel had been enlarged, but there was no mention of an organ being installed.^39^ Yet from 1849 an organist is listed annually among the hospital staff, first at a salary of £10 per annum, rising to £15 in 1853, £20 in 1859 and £25 in 1865. The puzzle is solved by searching in the General Ledger of 1840–53 (itself a challenge due to the layout of the evolving accounts) in which an Organ for the Chapel is listed in 1846, with the cost of £200. Annual payments of £5 5s to the organ builders JW Walker reveal the ongoing costs of maintaining the instrument.^40^

Further details tell us a little more about the state of instruments and how they might, therefore, have sounded. Accounts are littered with repairs, tuning and replacement parts or instruments, telling us a little about what was available for the band or for patient use. At other times we learn that instruments were not kept in good repair. At the West Riding Asylum in Wakefield, for example, the Commissioners noted in 1891 that the piano on the male side was ‘ancient’ and ‘unplayable’^41^ The Report of the following year recorded that the instruments had been repaired and tuned but ‘the organ could be still further improved’.^42^

Other raw material from the archives gives further perspectives on the ways in which music was introduced and the soundscape managed. As Sally Swartz has contended, beyond the realm of statistics ‘we are in a shadowland, where there is everything that cannot be counted’.^43^ Yet there is plenty to be gained also from minutes and committee papers, debates over financial decisions, correspondence between institutions and the views of individuals from the management. Returning momentarily to Bethlem, we can sense the discord in a Committee of Governors meetings in June 1843, when it was noted ‘And on considering the recommendation for “other musical instruments in addition to a Piano Forte” the same was declared to be lost; but upon a division being called for, there appeared to be 25 Governors in favour of the recommendation, and 23 against it, when it was declared from the Chair to have passed in the affirmative’.^44^ The same meeting approved ‘chairs table and carpet for the Workwomen’ but disallowed expenditure on a billiard table.^45^ The ledger confirms that a sum of £32 was spent on ‘Musical Instruments & Pianoforte’ in 1843.^46^ Combining these different formal records tells us not only what was spent on music, but that it was not universally valued by the Committee members.

## Musical Ephemera and Asylum Life

Asylum archives often include generous collections of music-related ephemera. Programmes and posters are generally the best source for the music actually performed, particular during the last few decades of the nineteenth century when a more concerted effort appears to have been made to catalogue and preserve examples. The existence of formal programmes may also point to the involvement of guests from outside the asylum, and certainly suggests attempts to put entertainments on a more formal footing. Sometimes programmes include the details of performers, giving an indication of the talents of internal staff as well as the extent to which visiting professionals and amateurs were engaged. An extensive collection in the Bethlem Hospital archive, for example, includes handwritten programmes from soirées and smoking concerts, which are unusual due to their miscellaneous and informal nature. Many of these include performer names, with a few also carrying pencil annotations either giving sketches of the performers or a critic’s view of the performance.

These small-scale concerts are testimony to the musical talents of many patients and staff, reflecting the middle-class nature of the patient body at Bethlem in the late-nineteenth century. Programmes in the Surrey History Centre relating to the Brookwood pauper asylum and the Holloway Sanatorium also give detail on the pieces performed and names of the performers. Like Bethlem, the middle-class institution of the Holloway Sanatorium supported frequent small-scale and informal events, while the larger pauper asylum at Brookwood focussed on formal events featuring the band, theatrical stagings and visiting performers. Pauper asylums rarely featured musical participation by patients, with the band restricted to male attendants and other performances provided by visiting performers, but the smaller occasions at private and charitable asylums were an opportunity for patients, staff and officers to mix.

The Brookwood archive includes a particularly valuable source for the year 1889, which details not only the musical content of weekly dances and performances, but the attendance figures for patients and staff, as well as the names of senior officers attending.^47^ Although formal reports often give a number or percentage of patients attending, it is rare to have this set out on a weekly basis. The particular inclusion of officers’ names shows that the senior medical officers were very regular supporters of the weekly entertainments, with almost all present each week. The full set of programmes for the year is testimony to the enormous repertoire of the Brookwood band, drawn from opera overtures and extracts, to dances based on popular tunes. The musical events recorded here allow us to build a picture of the asylum as a community, and the relationships fostered between patients, staff and management, as well as the ways in which music and entertainment were experienced.

The Brookwood archive also holds rare photographs of the asylum band, dating from the 1880s to 1890s. Although impossible to date fully, most of the photographs are at least partially annotated with names, allowing the particular involvement of individuals to be traced. Photographic evidence together with wage records show that many men remained in the employment of the asylum and in the voluntary band for long stretches of time, often over a decade.^48^ With little additional remuneration for their band duties, we can surmise that playing in the band was an enjoyable task, and with such regular and lengthy performance experience it is reasonable to suggest that the standard of such performers might have become quite considerable. These photographs also give a more detailed sense of the size and composition of asylum bands. Although referred to as a band, sometimes a brass band, photographs show a wider range of instruments including strings and wind. One photograph from the Wakefield archives from around the turn of the century shows a band of 37, including 21 strings: an ensemble more closely approximating an orchestra.^49^ In contrast, the Brookwood bands photographed during the last few decades of the nineteenth century ranged from just 10 to 21 musicians, with a double bass and clarinets the only non-brass instruments.^50^

As noted above, a small number of asylums have musical archives extant, although no longer held together with the documents and other elements of the institutional holdings. The music Edward Elgar composed during his time at Worcester lay untouched for many years, something of a skeleton in his compositional cupboard, until reconstructed and recorded in recent years by Barry Collett and members of the City of Birmingham Symphony Orchestra.^51^ The music is simple and could be described as naïve, using the most basic of dance forms and little of the orchestral and harmonic richness for which Elgar’s later became loved. Nevertheless, the jaunty melodies help conjure up images of the dances and fetes where they were heard. From the collection we learn much about the actual composition of the asylum’s band, the abilities of its players, and the kinds of repertoire that were probably most heard within the asylum’s walls.^52^

Another source is to be found in the vast collection of late-nineteenth and early-twentieth-century band music extant from the Norfolk County Asylum at Thorpe, near Norwich, now in the care of David Juritz, who performs from this repertoire together with colleagues from the London Mozart Players.^53^ Although the Norfolk collection has little composed specifically for the asylum, the parts similarly give an insight into the way music functioned, with a violinist leading the group and a piano part available to cover any missing harmonies. With differing instrumentations for each set of pieces, and some parts evidently used more than others, we might imagine the band members became adept at working from whichever parts and performers were at hand. Drawn together these sources give us a sense of the bands’ performances: a steady turnover of performers on basic pay, fluctuating instrumentation not always well-suited to the musical parts provided, a range of shapes and sizes varying between and within institution, attendants juggling the demands of the band with their busy day-to-day work, yet wide repertoires and varied programmes, regular performances and often the involvement of local professionals either in performing or leadership roles.

Together with the archives created for and about the management of the hospital, a second set of records relates more directly to the patients. Case notes form the bulk of many archival holdings from nineteenth-century asylums, simply because of their scale. Each patient was assessed on arrival, with two independent letters required to secure certification and admission. Closely monitored for several weeks at the start of their residence, they then tended to receive only monthly attention until discharge. Patient case notes have formed an increasingly important part of asylum history, with individual institutional studies contributing important insights into the ways in which patients were categorised and treated as well asthey ways in which they related to staff and maintained relationships with family and friends.^54^ Case notes tend to focus on the mental state of patients and the physical manifestations of this, together with any treatment administered.

There is little information on the patient’s activities, and as such, little further information about the uses of music to be gleaned from case notes. The few examples of musical activity I have identified reveal a divided picture: on the one hand, music in the form of playing the piano or joining in a dance could be indicative of a return to mental stability; on the other hand, singing or dancing in an uncontrolled manner was a sign of distress. Music in the case books appears as a symptom, rather than a form of treatment or therapy, perhaps because of the ways in which the case book notes reflect a snapshot of patient behaviour rather than a continuous narrative. The few mentions of music give us some of the only direct evidence of individual patients engaging with music, whether making music (in the widest sense), dancing or listening. They rarely go beyond a brief mention. However, the absence of regular accounts of patient engagement in music cast further light on the connections made between music and mental well-being in the formal reports. This confirms that music was not seen as a personal method of treatment or recovery, and remained a generalised attempt to entertain with associated healing properties. It also suggests that the relationship between music and health received very little empirical attention during the period, with few of the case records I have examined pointing to direct links between musical activity and patient wellbeing. There remains a disconnect between the positive ways in which music was portrayed in the formal Reports, and the scant mention of it in the case notes.

While containing little information about the therapeutic uses of music, case notes can certainly be used as evidence for the broader soundscape of the asylum, including the sounds of patients themselves. The sonic activity of patients was used as part of their diagnosis and ongoing assessment. ‘Noisy’ was regularly used as a description for patients, and included in the categories set out by Samuel Tuke in 1815, where ‘patients who are disposed to incoherent laughing and singing’ were among the first of three separate classes.^55^ Dolly MacKinnon’s work on the asylum soundscape reflects on the importance of a range of archival documents and artefacts for constructing a detailed sense of the sounds surrounding patients.^56^ Focussing on Australian case notes from the early twentieth century, MacKinnon notes ‘Language, sounds, noises and silence defined patients both socially and intellectually’, while ‘Ambiguous speech, sounds, silences, or noises demonstrated and defined the sonic transgressions of sounding sane, and performing madness’.^57^

Individual patients are also represented in the form of patient-produced material, including poems, artwork, letters and prose. Much of this material gives a sense of patients’ state of mind, but little firm information about their conditions or activities. However, some accounts printed after patients returned to outside world give a more lucid sense of what life was like, and these include valuable descriptions of music experienced within each institution. John Perceval’s narrative of his time at the Ticehurst private asylum during the early nineteenth century, for example, recounts that he was provided with a piano, which he recalled ‘peaceably striking … during the most part of the evening’.^58^ However, he also complained of the noise from other patients and servants ‘whistling, singing, fluting, fifing, fiddling, laughing, talking, running and even occasionally dancing in the passages and wrestling’.^59^ Herman Charles Merivale, also at Ticehurst, recalls ‘little evening parties for whist or music amongst “ourselves,” and a casual conjuror or entertainer from town to distract us sometimes for an evening’, as well as ‘little card-parties and musical evenings’ on the ladies’ side.^60^ Another form of evidence is available from the semi-fictitious account of ‘Mabel Etchell’ written by Charlotte Phillips, who spent time in the West Riding Lunatic Asylum. The account of a ball in this novel includes details of the timings and arrangement for the dancing, patients’ dress and demeanour, the dancing itself and the interactions between patients and members of the asylum’s management.^61^

One key exception within the genre of patient-produced material is the asylum magazine. Important examples were produced at both Bethlem Hospital and Holloway Sanatorium. These two institutions shared the innovation of asylum boarders: individuals volunteering to live within the asylum without formal committal, believing that their mental health could best be maintained within the structures and care of a semi-closed environment. The voluntary boarders were influential in both institutions developing social activities. Within the private, middle-class asylum environment patients were at greater liberty to organise social gatherings including musical evenings; many would have been familiar with this task and many brought with them belongings such as musical instruments. At Bethlem, for example, the patient Henry Francis Harding took on the role of sub-editor for the magazine *Under the Dome*, produced in a number of versions between about 1875 and 1930.^62^ The extant printed copies of the magazine, dating from its later incarnation between 1891 and 1930, carried critical reviews of concerts and other entertainments.

While Bethlem boasted a semi-professional orchestra including many of its senior officers, and therefore largely glowing accounts of its performances, the parallel magazine at the Holloway Sanatorium helps give a further indication of musical activity there. The St Ann’s Magazine, which was published in the 1890s, adopted a faintly mocking tone: although the band was evidently appreciated, one writer suggested ‘some members of the band try to play two tunes at the same time. We are not musical, but the effect seems to be as we indicate’.^63^ Where patient magazines are extant, they often carry complete programmes of events for the period under review, a key addition to the information available in formal reports and via collections of programmes and posters.

In searching for first-hand accounts of the music and entertainment experienced at nineteenth-century lunatic asylums, third-party newspaper articles provide important sources of information, including commentary on the responses of the patients and other guests. In the account of a ball at the Royal Edinburgh Asylum in Morningside, the author noted that the dance ‘proves not only harmless, but, by diverting their thoughts and senses from the exciting cause of their malady, is a relief and a benefit … the same patients who are often noisy and obstreperous in their ordinary abodes in the asylum, behave with the utmost decorum at the soirées’.^64^ Another account recorded the Asylum band performing in between theatrical numbers, by which they ‘acquitted themselves very satisfactorily in a number of pieces, which not only filled in the time, but seemed to afford great delight to many of the patients’.^65^ The writer concluded that it ‘was impossible to have been present without seeing that such relaxations are very useful, not only to the insane, but to all the parties concerned. The patients in particular manifested evident enjoyment’. The newspaper reports go some way to support the claims for music made by the Superintendents: in another account from Wells the author recalled ‘we noticed faces which were at first dull and vapid gradually lighting up beneath the genial influence of the holiday treat, and in two instances which at the outset were painfully pensive became relaxed in the end by positive laughter’, while another record of the same event concluded ‘the entertainments produced a very salutary influence on the unhappy inmates of the institution, who behaved a in a manner that would have done no discredit to a perfectly sane auditory’.^66^

Thus the combination of the formal and informal reports and records stemming from the asylum’s management, together with patient records and publications, begin to give a rounded picture of how music was organised, what was played, who was performing, the standards attained, and the involvement of patients in music making. The rosy pictures portrayed in many Annual Reports, and the encouragements of the Commissioners in Lunacy, which linked music to the asylum’s role in mental wellbeing, are tempered by the accounts in the patient magazines suggesting the band was far from a professional outfit; furthermore, patient case notes and narratives frequently allude to music as a disturbance or a sign of mental instability as well as a potential healer. The provision of pianos in many wards, supported by the Commissioners and portrayed as an important option for patient recreation and occupation by many Superintendents, appears less benevolent when matched with patient stories about incessant playing, the poor condition of instruments and midnight noise. Yet the independent newspaper reports support many aspects of the claims made for music, suggesting that some, if not all, of the patients, took comfort from and responded positively to the opportunity for music and dance.

## Imaginary Records and the Creative Historian

One of the silences examined by Anne J. Gilliland and Michelle Caswell concerns the ‘record-that-never-was’, particularly relevant to patient case notes which are thin on detail regarding the day-to-day activities of patients and their responses.^67^ While we have the overarching claims regarding the efficacy of music made by the senior medical and managerial officers, we rarely have individual case exemplars to back up their claims. This kind of day-to-day record is not generally part of the case note, but this makes it hard to assess whether the claims made for music were genuinely noticed in individual patients. Likewise, there are countless surviving examples of patient-created material, some of which has been published, but much extant in manuscript form in archive holdings. Very little of the unpublished material touches on day-to-day activity; it is largely concerned with arguing for a patient’s release in the context of unjustifiable incarceration. Published memoirs give far more detail on the running of the asylums and each patient’s experience, and often include reference to daily activities, routines, and the impact of a ball or hearing the band. Yet I have not identified any patient-produced material that refers to the positive influence of music in the same way as it is described in many formal reports.

How do we interpret the claims of Medical Superintendents in this vacuum? Ought we to imagine that those references would exist, had that kind of detail been recorded in patient notes and more generally in patient-produced material? Or do we ‘follow the money’, noting that Superintendents were obliged to justify their decisions, particularly regarding any expenditure potentially perceived as excessive, to the board of Visitors, and that therefore the link between music, health and recovery was an important one to emphasise regardless of the actual experiences of patients. My interpretation has been the latter one—I have not, in this case, imagined these records—but it is instructive to consider the potential for alternative histories had record-keeping been different and other evidence created. The relevant archival imaginary in this case, the evidence of music’s power in treating mental illnesses, is also one sought after by generations. The Victorians were able to draw on a long literature of publications expounding music’s role in treating lame animals, revivifying depressed females and calling reluctant men to battle or other feats. The twentieth-century academic, likewise, is often eager to find scope for the impact of their research through uncovering new information about the close links between music and well-being. The lure of imagining further details to add to the claims made in formal reports needs careful balance with the historical context of those documents. Some of these ‘imaginaries’ may yet be discovered through further research; my own work has been far from comprehensive. Yet the thought process of considering archival content, historical context and research motivations provides much food for thought.

More generally, despite the rich vein of information on the music available in asylums, and our ability to draw on information regarding the ways in which it was performed and even some comment on the standards of the performers, we have little to tell us about the ways in which music was experienced. The patient magazines of the Holloway Sanatorium and Bethlem Hospital include patient accounts of concerts, but accounts of the listeners are conspicuously absent. Newspaper accounts tell us of patients responding in positive ways to the music of the dance, theatrical or concert, but these do not explore the individual circumstances of patients. To these sets of materials we can also add the published writings of asylum medics, some of which do explore the ways in which music and health interacted within the world of mental health. Two papers given in 1894 by William Ireland and Richard Legge explored the particular musical abilities of patients with mental disorders, while Herbert Hayes Newington, from the private asylum at Ticehurst, attempted in 1897 to explain the mental processes which accompanied musical performance.^68^

Putting the work of an asylum and its management in context requires tracing the individuals involved beyond their input at a single institution. Nineteenth-century alienists often moved from one institution to another in the process of gaining experience and promotions, and it was not unusual for medical superintendents to move between the public and the private sector; indeed, some managed a private asylum at the same time as holding a post at one of the pauper institutions. Many published their views and experiences, whether as part of an overview of the treatments of mental illnesses, or a detailed paper in one of the new journals dedicated to their work.

In some cases, the theory expounded in published material needs some triangulation with the details of practice in institutional archives. In the case of moral management and music, for example, many writers explained the importance of moral treatment being tailored to the needs of individual patients, but in practice little of this is evident. The Scottish alienist William A.F. Browne gave two examples of music being used in the asylum during his 1864 lecture to a Medical Psychology class at the Crichton Institution in Dumfries. First, he noted ‘Watch an assemblage of lunatics while national or cheerful airs are played; and it becomes palpable that though dead to all else, they are alive to sweet and familiar sounds’.^69^ Second, he related ‘A lady after hearing Scotch music retired to bed degraded, mute, fatuous; she arose next morning and remained permanently of right and rational mind, quietly remarking to the physician, that “The banks and braes o’ bonny Doon had awakened her.”’^70^ Browne illustrated the widespread popularity of music within asylums, recording ‘We are familiar with all this; classes, concerts, bands, &c., form one of the embellishments of every asylum’.^71^ His generalisation about the positive effect of music is similar to many statements recorded in both published theoretical material and the Reports and records of archives. His story about the lady fits into a long tradition of anecdotal evidence about the power of music, though there are few other tales of similar individuals within nineteenth-century asylums.

Taken together, the rich holdings of asylum archives can begin to reveal something of the asylum soundscape, drawn from formal and informal music-making, from the proficient professional performer to the noise signifying mental instability and patient distress. The variety of music and noise evidently went well beyond the ordered impression of music given in the formal reports. How can we bolster these soundscapes further with modern resources outside the archives, and how might these be incorporated into the historical narrative?

## Re-sounding the Asylum: Music and Historical Perspective

Of course it is not just the archives that help us to build a picture—and soundscape—of asylum sites during their heyday in the nineteenth century. Many of the sites are still accessible and located nearby the relevant archives, adding a practical and intellectual benefit to visiting archives in person. The majority of pauper asylums closed during the second half of the twentieth century, with only a few now used for health-related work, either as NHS sites (such as part of the Norfolk Asylum, and the Cambridgeshire Asylum at Fulbourn) or private mental hospitals (such as the Priory at the former Ticehurst hospital). This allows the archive historian to gain a sense of the scale of asylum buildings and surroundings which, together with historical maps, can reveal much about the geography and built heritage of the asylum project. With many asylum sites converted into luxury accommodation, access to the locations of musical entertainment is limited. The recreation halls at the West Riding Asylum in Wakefield and the Cambridgeshire County Asylum in Fulbourn are extant. The enormous and solid bulk of the former, once the comically-titled ‘Theatre Royal, Stanley-cum-Wrenthorpe’, reflects the asylum’s population of over 1,000 patients in the late nineteenth century as well as the central place of music and theatricals at that asylum from the mid-nineteenth century. The quirky octagonal chapel at the Norfolk asylum remains, though converted into accommodation, while the grand, late-Victorian chapel at the Holloway Sanatorium stands as a monument to its founder’s lofty aims. Most former asylum complexes now stand relatively silent, giving little indication of the lively strains of music and noise of patient life that once prevailed.

Being able to visualise the sites, particularly their enormous scale, helps to concretise the information held in archive documents, such as the physical work of getting patients from one end of the site to the other for recreational activities, as well as the austere nature of the buildings. Buildings can help amplify information on the kinds of activities taking place, and the ways in which they operated: what kind of a sized space was used for several hundred patients at a dance, for example; what was the size and acoustic like for a church choir of 16; how many patients might the chapel have held for concerts and services? Many of the purpose-built chapels and recreation halls, constructed to develop the asylums as societies-in-miniature as part of patient rehabilitation, are still extant, though I have not visited any generally open to the public. Whether converted to luxury housing, in use as a gym or church, repurposed as NHS offices or left to dereliction, the former sites of musical—and other—activity are now radically altered in their soundscapes. The importance of place and experience have begun to receive attention in archive studies: both Gesa E. Kirsch and Liz Rohan draw on personal experience in their book *Beyond the Archives* to examine the importance of ‘closeness’ to the historical subject, both intellectually and emotionally, and physically through exploration of place and location.^72^ Modern experiences, such as walking the old asylum buildings and grounds, hearing music reconstructed, patched together with the extant evidence explored here, begin to allow for a creative and imaginary sense of the Victorian asylum soundscape.

Some more recent videos taken of the nineteenth-century asylums featured in my study illustrate the important role of music in the ongoing presentation and reputation of asylum institutions. One rare example of footage from Powick asylum near Worcester eschews a musical background, remaining silent except for footage of recreational piano playing at the point where the film in its modern incarnation is split in two.^73^ Instead, the voices of the narrator, patients and staff are accompanied only by the minimalist soundscape of muffled voices, clattering tea cups and wheelchairs on the move. In contrast, a film made using footage from the Stanley Royd Hospital, formerly the West Riding Lunatic Asylum, after its closure in 1995 uses the slow, repetitive build-up of notes and rhythms from the 2009 track ‘Forget This Ever Happened’ from the artist Mr Meeble.^74^ Drawing together minimalist influences with electronica to effect a meditative aura, the musical accompaniment perhaps reflects the status of the asylum post closure. No longer a working environment, the ghosts of its former residents, staff and activities are echoed in the eerie sounds present in Mr Meeble’s track. A further contrast is offered by a more recent programme from the BBA, where the opening of the film is accompanied by active, racing music, followed by silence during original footage.^75^ Is this part of the BBC’s homage to the past? To be sure, leaving the soundtrack behind avoids layering another aspect of interpretation onto the footage for viewers, but historical film of this age is hardly neutral.

Viewing historical images of asylums—whether film or photographs—or visiting the sites almost certainly carries a different aural experience from that of the institutions during their Victorian heyday. While much can be captured in film or photograph, the place and experience of sound is much harder to draw into the modern era. Without a soundtrack, silenced asylums assume the mysterious and creepy aura that imbues much of their reputation, especially since the closure of institutions and their dereliction or repurposing. A similar effect is achieved with a soundtrack that emphasises emptiness or paranormal through electronic sounds or repetitive notes. At the same time, using some of the music we know to have been played in nineteenth-century asylums does a disservice to those who lived and worked there, and for whom life was undoubtedly tough. The jaunty tunes of Elgar’s band music for the Powick Asylum, or the simple ballads and airs of the miscellaneous concerts of the pauper asylums and soirées of the private institutions were, we know, moments of release in an otherwise monotonous existence. The musical portions represent only part of the wider asylum soundscape, and recreating them usually fails to take into account the variable availability and ability of performers, the state of instruments, and the ambient noise of an audience.

## Conclusions

Through investigating musical and soundscape silences, we are pushed to use every facet of the archive and beyond, to reconstruct, and to imagine, in order to begin to re-conceive not only what music was heard but how it was experienced. We follow individuals—staff members of all ranks, and patients—and put ourselves in their shoes as best as we can in order to understand the potential impact of music. Examining the locations of music and entertainment offers new perspectives on the ways in which moral management in the asylum was interpreted, the relationships between staff and patients, management and visitors, institution and external community. Because of the literal silences of the archive, a musicological investigation draws on these resources to look anew at the ways we can research history. Delving into the silences surrounding the musical and aural experience of the nineteenth-century asylum helps to highlight other, metaphorical, silences concerning personal experience and day-to-day life in the asylum. It prompts new consideration of the wealth of patient material and case notes, as well as an examination of the values and practices of medical and lay management. This kind of a model provides new directions and approaches for archival research, as well as new points for consideration by curators, archivists and historians. A deeper look into the musical soundscape of the asylum further helps to tease apart some of the meanings of music during the period: its capacity for mental and moral well-being, its social and cultural role in the fabric of life, its place in creating a ‘homeliness’ or sense of normality, and its effect on the individual.

The move towards ‘history from below’, where asylums and health are concerned, meets further challenges in the contexts of music and the soundscape, with such a paucity of information either directly from patients or concerning the patient experience. Careful handling of official documents, triangulated with the scarce testimony from former patients or via patient magazines, mean we have a sense that the musical experience was often patchy, at best. Despite the links made between music and mental well-being, we can be sure that at least part of this argument was linked to the need to present a case for the asylum’s successful management and use of public funds. And where documents record the purchase of instruments and successful formation of bands and choirs, elsewhere it is clear that resources were often in poor shape and standards very variable. A thorough examination of the archives balances the pictures painted in the annual reports with the realities of finances, resources and people. This further acts as a cautionary tale in other areas of asylum research and beyond, highlighting the potential gap between the values and ideals expressed in publications and reports, and the actual experience of management, staff and patient.

How, then, do we go about getting our sense of how music was, how it actually sounded and how it was experienced? Despite the wealth of different sources, it is necessary to get past the sanitised accounts, data, lists of repertoire, names and instruments, buildings and spaces. In addition, how do we take account of the mental states of the patients: some mentally ill, some frightened or frustrated by their situation, and all from a distant time and space meaning their experience of music must have been quite different from our own. It is in this context that imagined silences and leaps of imagination might come to the fore. Putting together the information from the resources I have outlined gives us a good foundation for imagining the soundscape and musical environment of the nineteenth-century asylum, but perhaps we are still unable to access the ultimate musico-historical questions: how was music experienced in the past, how did it sound and what did this mean for the listeners and performers of distant times?

The idea of archival silence as a metaphor has become useful for scholars of critical archive studies, but presents particular meanings for musicologists engaging in archival work. Can the work of musicologists, conversely, offer critical tools for archivists, scholars of the archive and historians? The silences investigated and probed here are an important reminder of the gaps inherent in the archive and the historical narratives that can, and can’t, be drawn from deep engagement with archive material. The silences posited by scholars of archives are metaphorical, but also literal, reflecting the multifaceted dimensions and sensory input lost through the capture of data and material in written or pictorial form. Turning the silences of the archive upside down to interrogate historical noise, music and sound contributes to a wider perspective on the ways in which history might find a deeper and more sensory approach. This can find practical applications in the types of films I have discussed, historical documentaries, museum displays and written histories. Thus while archives themselves are likely to remain silent, it is clear that noise and music add a vital dimension to our understanding and presentation of the past.

Musicology often deals with modern-day silences and the need to make imaginative leaps in order to explore historical musics and musical experiences. Music is bound in time, fleeting, and its experience only translated into the texts and documents that are preserved. Thus much of musicological-historical study is focussed on music and musical experience as narrated second hand, or on the peripheral aspects of the music business, reception studies or music education. The call to ‘return to the music itself’ is a frequent trope, but at the same time the contingent and partial nature of the musical score or text is similarly recognised. The role of music in the history of medicine is equally hard to pin down, with both the traces of musical performance and clues to its impact and influence providing starting points for constructing a new understanding of music’s place in mental health history. Exploring the musical soundworld of the asylum offers a new dimension for studies of mental health in this historical context, but also prompts new ways of investigating the social history of medicine more broadly, through new perspectives, documents and materials. It highlights, once again, the difficulty of accessing the patient experience and the problems of narrative voice and perspective. Considering sound and music further prompts engagement with, and critique of, the modern experience of buildings and film. The historical distances involved in musicology make the need for imagination and creativity particularly clear, but the gaps and silences in other areas of history are no less real.

## Footnotes

1 On the contextual history of music, see Trevor Herbert, ‘Social History and Music History’ in Martin Clayton, Trevor Herbert and Richard Middleton, eds, *The Cultural Study of Music: A Critical Introduction* (New York and London: Routledge, 2003), 146–56.

2 Key texts include Catharine Coleborne and Dolly MacKinnon, eds, *‘Madness’ in Australia: Histories, Heritage and the Asylum* (St Lucia, QLD, Australia: The University of Queensland Press, 2003); Stephen Cherry, *Mental Health Care in Modern England: The Norfolk Lunatic Asylum St Andrew’s Hospital, 1810-1998* (Boydell Press, 2003); Leonard Smith, *Cure, Comfort and Safe Custody: Public Lunatic Asylums in Early Nineteenth Century England* (Leicester UP, New York, 1999); Andrew Scull, *The Most Solitary of Afflictions: Madness and Society in Britain, 1700-1900* (New Haven and London: Yale University Press, 1993); Mackenzie Charlotte, *Psychiatry for the Rich: A History of Ticehurst Asylum* (Wellcome Institute Series in the History of Medicine, London and New York, Routledge, 1992); and Anne Digby, *Madness, Morality and Medicine: A Study of the York Retreat, 1796-1914* (Cambridge: Cambridge University Press, 1985). More recent publications include Kathryn M. Burtinshaw and John R.F. Burt, *Lunatics, Imbeciles and Idiots: A History of Insanity in Nineteenth-Century Britain and Ireland* (Barnsley: Pen and Sword, 2017); Jennifer Wallis, *Investigating the Body in the Victorian Asylum: Doctors, Patients and Practices* (Cham: Palgrave Macmillan, 2017); Louise Hide, *Gender and Class in English Asylums, 1890-1914* (Basingstoke: Palgrave Macmillan, 2014); and Anna Shepherd, *Institutionalizing the Insane in Nineteenth-Century England* (London: Pickering and Chatto, 2014).

3 See Jennifer Wallis, *Investigating the Body in the Victorian Asylum: Doctors, Patients, and Practices* (Cham: Palgrave Macmillan, 2017). Wallis’s book is innovative in its organisation by parts of the body, but the ear, hearing and aspects of aural experience are noticeable by their absence. MacKinnon’s work includes ‘ “Hearing madness”: The soundscape of the asylum’ in Catharine Coleborne and Dolly MacKinnon, eds, *‘Madness' in Australia*, 73–82; ‘Music, Madness and the Body: Symptom and Cure’ in *History of Psychiatry*, 2006, XVII, 9–21; and ‘Hearing Madness and Sounding Cure: Recovering Historical Soundscapes of the Asylum’ in *Politiques de communication* Special Issue 1, 2017, 77–106.

4 John Conolly, *The Treatment of the Insane Without Mechanical Restraints* (London: Smith, Elder & Co., 1856), 6. See also Conolly, *An Inquiry Concerning the Indications of Insanity, with Suggestions for the Better Protection and Care of the Insane* (London: John Taylor, 1830) and *The Construction and Government of Lunatic Asylums and Hospitals for the Insane* (London: John Churchill, 1847).

5 For an overview of moral management, see Leonard Smith, *Cure, Comfort and Safe Custody: Public Lunatic Asylums in Early Nineteenth Century England* (Leicester University Press, New York, 1999), 187–94. See also Heidi Rimke and Alan Hunt, ‘From Sinners to Degenerates: The Medicalization of Morality in the 19th Century’ in *History of the Human Sciences*, 2002, XV, 59–88. On the arts in asylums, see in particular Maureen Park and Robert Hamilton, ‘Moral Treatment of the Insane: Provisions for Lifelong Learning, Cultural Engagement, and Creativity in Nineteenth-century Asylums’ in *Journal of Adult and Continuing Education*, 2010, XVI, 100–13. Music’s links with medicine and health can be traced back to the Ancient Greeks; more recently numerous publications had reproduced anecdotes relating to music’s power, including John Taylor, *The Power of Music, in which is Shown, by a Variety of Pleasing and Instructive Anecdotes, the Effects it has on Man and Animals* (London: J. Harris, 1814) and J. G. Millingen, ‘Medical Powers of Music’ in *Curiosities of Medical Experience*, 2nd edition (London: Bentley, 1839), 88–96.

6 See, for example, Stephen Cherry and Roger Munting, ‘‘Exercise is the Thing’? Sport and the Asylum c.1850-1950’ in *The International Journal of the History of Sport*, 2005, XXII, 42–58 and Juliet Foster, ‘Performance in Bethlem, Fulbourn and Brookwood Hospitals: A Social Psychological and Social Historical Examination’ in Anna Harpin and Juliet Foster, eds, *Performance, Madness and Psychiatry: Isolated Acts* (Basingstoke: Palgrave Macmillan, 2014), 42–62.

7 See Rosemary Golding, *Music and Moral Management in the Nineteenth-Century English Lunatic Asylum* (Cham: Palgrave Macmillan, 2021), 60–62, 217–218.

8 George Man Burrows, *Commentaries on the Causes, Forms, Symptoms, and Treatment, Moral and Medical, of Insanity* (London: Thomas and George Underwood, 1828), 706, and John Conolly, *The Construction and Government of Lunatic Asylums, and Hospitals for the Insane* (London: Churchill, 1847), 55. Conolly recommended supplying or procuring pianos and other instruments, and organising music evenings and dancing parties.

9 The term ‘band’ was used initially to refer to a small group of performers, usually predominantly brass instruments, with clarinets and flutes. Later in the nineteenth century some asylum bands took the form of a full wind band with double bass, while others comprised 30–40 performers, including strings. The asylum band at the West Riding Asylum was unusual in being formed from string instruments. A brass band was originally established in the late 1850s, but swiftly replaced with strings, considered more appropriate for the new recreation hall. By 1863 an orchestra of 22 players was in place. See ‘Quarterly Report to the Committee of Visitors’ 28 April 1859 in Medical Director’s Journal 1858–1867 [West Yorkshire Archive Service C85/1/13/1] and Annual Report [C85/1/12/2] 1863–4, 17.

10 The *Journal of Mental Science* published several articles on music in the 1890s, while publications such as Daniel Hack Tuke’s *A Dictionary of Psychological Medicine* also include mentions of music (see ‘Treatment (General)’, 1315–16). On Frederick Kill Harford and the Guild of St Cecilia see William B. Davis, ‘Music Therapy in Victorian England’ in *Journal of British Music Therapy*, 1988, II, 10–16.

11 It is notable, for example, that the inclusion of pianos as standard in asylum wards and nurses’ accommodation towards the end of the nineteenth century moved in parallel to the mass ownership of pianos among the middle classes from the 1870s. See Cyril Ehrlich, *The Piano: A History* (rev. edn. Oxford: Clarendon Press, 1990), 92–94 and 98–100.

12 On music in private asylums, see Golding, *Music and Moral**Management*, 24–25, 59–61, 237–315. Whereas men and women patients at the pauper asylums had the same exposure to music, at the dance and chapel, other forms of music in the smaller private and charitable asylums were more heavily gendered: women attended soirées of instrumental and vocal music while men held ‘smoking concerts’, featuring bawdy songs, jokes and acts.

13 Local journalists were often invited to visit the asylum during a dance or fete, at which external dignitaries were also treated to a sanitised view of the institution’s best-behaved patients. The asylum ball also features in numerous examples of novels, in which the image of the large-scale dance offers a colourful focus. Among the more famous examples is to be found in Charles Dickens, *The Old Curiosity Shop*, Vol. II [1840[ (New York: Cosimo Classics, 2009), 175. See also Sander L. Gilman, *Disease and Representation: Images of Illness from Madness to Aids* (Ithaca: Cornell University Press, 1988), 48; and Dolly MacKinnon, ‘Music, Madness and the Body: Symptom and Cure’ in *History of Psychiatry*, 2006, XVII, 9–21, 12.

14 See, for example, discussion in Michelle Caswell, Harrison Cole, and Zachary Griffith, ‘Images, Silences, and the Archival Record: An Interview with Michelle Caswell’ in *disClosure: A Journal of Social Theory*, 2018, XXVII, 21–27. DOI: 10.13023/disclosure.27.04, 27.

15 Anne J. Gilliland and Michelle Caswell, ‘Records and their Imaginaries: Imagining the Impossible, Making Possible the Imagined’ in *Archival Science*, 2016, XVI, 53–75. DOI: 10.1007/s10502-015-9259-z, 55.

16 See Michelle Caswell, Ricardo Punzalan, and T-Kay Sangwand, ‘Critical Archival Studies: An Introduction’ in “Critical Archival Studies,” eds. Michelle Caswell, Ricardo Punzalan, and T-Kay Sangwand. Special issue, *Journal of Critical Library and Information Studies*, 2017, I. DOI: 10.24242/jclis.v1i2.50, 1.

17
*Ibid*., 69.

18 See, in particular, Roy Porter, ‘The Patient's View: Doing Medical History from Below’ in *Theory and Society*, 1985, XIV, 175–98.

19 Robin G. Collingwood, *The Idea of History* (Oxford: Oxford University Press, 1946, repr. 1994).

20
*Ibid.*, 228.

21
*Ibid*., 242–3.

22 See the ‘Sounding Victorian’ project led by Phyllis Weliver and Sophie Fuller (http://www.soundingvictorian.org/) (last accessed 17 October 2020; this website is currently unavailable).

23 See Helen Barlow and Trevor Herbert, ‘Introduction, Listeners in Music History: Studying the Evidence’ in *Nineteenth-Century Music Review*, 2020, XVII. doi:10.1017/S1479409819000582, 1–15.

24 Ian Biddle and Kirsten Gibson, ‘Introduction’ in *Cultural Histories of Noise, Sound and Listening in Europe, 1300–1918* (Abingdon: Routledge, 2017), 1.

25 Lawrence Kramer, *Musical Meaning: Toward a Critical History* (Berkeley and Los Angeles: University of California Press, 2001), 9.

26 Michelle Caswell, Ricardo Punzalan and T-Kay Sangwand, ‘Critical Archival Studies: An Introduction’ in Michelle Caswell, Ricardo Punzalan, and T-Kay Sangwand, eds, ‘Critical Archival Studies’, Special Issue, *Journal of Critical Library and Information Studies*, 2017, I. DOI:10.24242/jclis.v1i2.50

27 John M. Picker, *Victorian Soundscapes* (Oxford: Oxford University Press, 2003), 3–4.

28 To be sure, the soundscape went well beyond the realms of music, to include day-to-day noises as well as the individual imaginings of asylum patients; see Victoria Patton, ‘Voices from the Nineteenth–Century Asylum’ https://understandingvoices.com/exploring-voices/a-history-of-voices/voices-from-the-nineteenth-century-asylum. On the soundscape in Australian Asylums, see Dolly MacKinnon, ‘‘Hearing Madness’: The Soundscape of the Asylum’ in Catharine Coleborne and Dolly MacKinnon, eds, ‘*Madness' in Australia*, 73–82 and ‘Hearing Madness and Sounding Cure: Recovering Historical Soundscapes of the Asylum’ in *Politiques de communication*, 2017, 77–106.

29 See Barry Collett, ‘A Strange Apprenticeship: Elgar at Powick’ in *The Elgar Society Journal*, 1997, 284–95 and ‘Elgar’s Music for Powick Asylum’ in *The Elgar Society Journal*, 2013, XVIII, 53–54.

30 On Hanwell, for example, see Akihito Suzuki, ‘The Politics and Ideology of Non-Restraint: The Case of the Hanwell Asylum’ in *Medical History*, 2012, XXXIX, 1–17.

31 For a full study of this period see Michael Anthony Finn, *The West Riding Lunatic Asylum and the Making of the Modern Brain Sciences in the Nineteenth Century* (unpublished PhD thesis, University of Leeds, 2012).

32 The most comprehensive history is to be found in Jonathan Andrews, Asa Briggs, Roy Porter, Penny Tucker and Keir Waddington, *The History of Bethlem* (Abingdon: Routledge, 1997).

33 Overviews of these two institutions can be found at Anne Digby, *Madness, Morality and Medicine: A Study of the York Retreat, 1796–**1914* (Cambridge: Cambridge University Press, 1985) and Anna Shepherd, *Institutionalizing the Insane in Nineteenth-Century England* (London: Pickering and Chatto, 2014).

34 The most comprehensive survey of private lunatic asylums remains W. L. Parry’s Jones’s *The Trade in Lunacy: A Study of Private Madhouses in England in the Eighteenth and Nineteenth Centuries* (London: Routledge and Kegan Paul, 1972). The earlier period is now revisited in detail in Leonard Smith’s *Private Madhouses in England, 1640–1815: Commercialised Care for the Insane* (Basingstoke: Palgrave Macmillan, 2020). Numerous institutions are given detailed consideration in individual publications.

35 The Committee of Visitors was often drawn from the County or City Magistrates and Justices, and represented the areas covered by the Asylum.

36 On the Commissioners in Lunacy, see D. J. Mellett, ‘Bureaucracy and Mental Illness: The Commissioners in Lunacy 1845-90’ in *Medical History*, 1981, XXV, 221–50.

37 Asylum attendant staff were regularly drawn from the military, as the work required a high level of strength and fitness as well as discipline. This meant that some came with musical training; ability to contribute to music and sport was an element in appointing attendants at some asylums in the later-nineteenth century. See, for example, an application form for a male attendant contained in Worcestershire County and City Pauper Lunatic Asylum Archive, Worcestershire Record Office B499:9/10127/3 Salaries and Wages April 1893–March 1898. Most attendant and nursing staff lived on-site; other labourers might be drawn from the local community. The families of asylum officers were also important; wives and unmarried sisters of the Medical Superintendent, Chaplain and others were often involved in the institution’s role as a community.

38 1843 General Report for the Royal Hospitals of Bridewell and Bethlem, 49.

39 1845 Physicians’ Report for Bethlem, 10.

40 Bethlem Archive GRD-05 General ledger 1840–53, 412.

41 Report 1891, 36.

42 Report 1892, 33.

43 ‘Asylum Case Records: Fact and Fiction’ in *Rethinking History*, 2018, 22, 289–301, 292.

44 Bethlem Archive BCB-28: Minute book April 1841–November 1854, 26 June 1843.

45
*Ibid.*

46 GRD-05 General ledger 1840–53, 406: General Accounts 1843.

47 Surrey History Centre 3043/1/11/2/2.

48 Asylum bands were restricted to men; women musicians were involved in chapel music (usually playing the organ or harmonium rather than singing), informal chamber music concerts or soirées at private or charitable institutions, and, later in the century, in the development of musical theatrical productions such as those performed at the West Riding Asylum. Women musicians were drawn from among the nursing staff, the wives and daughters or asylum officers, and sometimes from the local community. Women were also among the visiting performers hired by asylums as part of regular entertainment.

49 West Yorkshire Archive Service C85/1402.

50 Surrey History Centre Photograph Album 3043/1/20/7 and 3043/10/2/7.

51 ‘Edward Elgar: Music for Powick Asylum’ [Innovation Chamber Ensemble, Barry Collett] [Somm: SOMMCD 252].

52 The detail of the music has been examined in Barry Collett, ‘A Strange Apprenticeship: Elgar at Powick’ in *The Elgar Society Journal*, 1996, IX, 284–95.

53 Juritz discussed the collection in ‘The Asylum Band’ broadcast on radio 4, 20 June 2006.

54 A key text is Roy Porter, ‘The Patient's View: Doing Medical History from Below’ in *Theory and Society*, 1985, XIV, 175–98. Recent publications examining the methods of patient-centred history include Brendan D. Kelly, ‘Searching for the Patient’s Voice in the Irish Asylums’ in *Medical Humanities*, 2016, XLII, 87–91; and Angela McCarthy *et al.*, ‘Lives in the Asylum Record, 1864 to 1910: Utilising Large Data Collection for Histories of Psychiatry and Mental Health’ in *Medical History*, 2017, LXI, 358–79.

55 Samuel Tuke, *Practical Hints on the Construction and Economy of Pauper Lunatic Asylums* (York: William Alexander, 1815), 18.

56 Dolly MacKinnon, ‘Hearing Madness and Sounding Cures: Recovering Historical Soundscapes of the Asylum’ in *Politiques de communication*, 2017, 77–106.

57
*Ibid*., 88–89.

58 John Perceval, *A Narrative of the Treatment Experienced by a Gentleman, during a State of Mental Derangement; Designed to Explain the Causes and the Nature of Insanity* (London: Effingham Wilson, 1840), 246.

59
*Ibid*., 229.

60 Herman Charles Merivale, *My Experience in a Lunatic Asylum, by a Sane Patient* (London: Chatto and Windus, 1879), 8, 149.

61 Mabel Etchell, *Ten Years in a Lunatic Asylum* (London: Simpkin, Marshall, 1865), 334–38.

62 Sarah Chaney, ‘‘No “Sane” Person Would Have Any Idea’: Patients’ Involvement in Late Nineteenth-century British Asylum Psychiatry’ in *Medical History*, 2016, LX, 37–53. On the Bethlem magazine see catalogue listing Under the Dome https://archives.museumofthemind.org.uk/UTD.htm (last accessed 16 June 2022).

63
*St Ann’s Magazine* Christmas 1896, 36.

64 Anon., ‘A ball at a lunatic asylum’ in *Chambers Edinburgh Journal* 101 (6 December 1845), 354.

65 Anon., ‘Amateur Theatricals at the County Asylum, Wells’ in *The Western Flying Post*, Yeovil, 24 January 1865. I am grateful to Peter Jaggard of the Friends of Mendip Hospital Cemetery for sending me this text.

66
*Taunton Courier and Western Advertiser*, 6 January 1864, 6 and *Shepton Mallet Journal*, 8 January 1864, 4. I am grateful to Peter Jaggard for this further reference.

67 Anne J. Gilliland and Michelle Caswell, ‘Records and their Imaginaries: Imagining the Impossible, Making Possible the Imagined’ in *Archive Science*, 2016, XVI, 56.

68 Richard Legge, ‘Music and the Musical Faculty in Insanity’ in *Journal of Mental Science*, 1894, XL, 368–75; William Wotherspoon Ireland, ‘On Affections of the Musical Faculty in Cerebral Diseases’ in *Journal of Mental Science*, 1894, XL, 354–67; and Herbert Hayes Newington, ‘Some Mental Aspects of Music’ in *Journal of Mental Science*, 1897, 704–23.

69 William A. F. Browne, ‘The Moral Treatment of the Insane’ in *British Journal of Psychiatry*, 1864, X, 309–37, 332.

70
*Ibid.*

71
*Ibid.*

72 Gesa E. Kirsch and Liz Rohan, *Beyond the Archives: Research as a Lived Process* (Southern Illinois University Press, 2008), 5.

73 ‘Powick Hospital Powick Near Worcester 1968 Part 1’ and ‘Powick Hospital Powick Near Worcester 1968 Part 2’ extracted from *World in Action* (1968), available at https://www.youtube.com/watch?v=UzjeBaBFWqw&feature=emb_rel_end and https://www.youtube.com/watch?v=ZJU4X60ce30 (last accessed 16 June 2022).

74 Mark Davis, ‘Stanley Royd, Former Pauper Lunatic Asylum Wakefield’ available at https://www.youtube.com/watch?time_continue=462&v=0Luut0g4Mmw&feature=emb_logo (last accessed 16 June 2022).

75 The Open University/ BBC, ‘Mental: A History of the Madhouse’, 9 February 2014, available at https://www.youtube.com/watch?v=Z0vuheEKMmI (last accessed 16 June 2022).

## Notes

Rosemary Golding is a Senior Lecturer in Music at the Open University, where she has worked since 2009. Her research covers areas of institutional and cultural history of music in nineteenth-century Britain, including publications on music in academia, music education and the music profession. Golding's current research draws on her long-standing interests in the relationship between music and health, and in historical perceptions of music's health-giving properties. Her current project is focussed around music in British asylums during the nineteenth century and she is preparing a monograph on this subject, drawing on a rich body of archive material. Her research blog is available at: https://musichealthandhappiness.wordpress.com/

